# Microbial BioRemediation Database: A Comprehensive Database of Genes Involved in Microbial Bioremediation Processes

**DOI:** 10.1002/mbo3.70215

**Published:** 2026-01-27

**Authors:** Silvia Petraro, Chiara Tarracchini, Leonardo Mancabelli, Gabriele Andrea Lugli, Francesca Turroni, Marco Ventura, Christian Milani

**Affiliations:** ^1^ Laboratory of Probiogenomics, Department of Chemistry, Life Sciences, and Environmental Sustainability University of Parma Parma Emilia‐Romagna Italy; ^2^ Microbiome Research Hub University of Parma Parma Emilia‐Romagna Italy; ^3^ Department of Medicine and Surgery University of Parma Parma Emilia‐Romagna Italy

**Keywords:** bioremediation, database, enzyme, microbial biodiversity, pollution

## Abstract

Environmental pollution from a wide range of compounds poses serious ecological and health risks. While bioremediation offers a promising solution, its application is limited by fragmented genomic resources and unsatisfactory understanding of microbial biodegradation pathways. Here, we developed the *Microbial BioRemediation* (*MBR*) database, freely accessible at https://probiogenomics.unipr.it/cmu, a comprehensive and manually curated repository comprising over 643,351 bacterial protein sequences associated with the degradation of 564 pollutant compounds across 25 chemical classes. Optimized for both genomic and metagenomic analyses, the *Microbial BioRemediation* database enables high‐resolution functional and taxonomic profiling of microbial communities and individual bacterial strains. Validation using public genome and metagenome datasets from contaminated environments confirmed the database ability to detect both conserved and environment‐specific biodegradation functions. Its application to host‐associated microbiomes further confirmed the suitability of *MBR* for assessing how environmental exposures shape microbial catabolic potential across ecological contexts. The *MBR* database thus serves as a strategic tool for the early‐stage identification and prioritization of microbial candidates for bioremediation. By enabling the in silico selection of key microbial taxa and enzymatic functions, it supports a rational pipeline that progresses toward targeted in vitro validation and experimental characterization. This integrative approach facilitates development of next‐generation, tailored strategies for the remediation of complex polluted ecosystems.

## Graphical abstract

We present the *Microbial BioRemediation* (*MBR*) database, a curated resource of over 642,000 bacterial protein sequences involved in the degradation of 564 environmental pollutants. By enabling rapid functional profiling of microbial genomes and communities across both environmental and host‐associated ecosystems, *MBR* supports the identification of key biodegradation functions and microbial candidates for targeted bioremediation strategies.

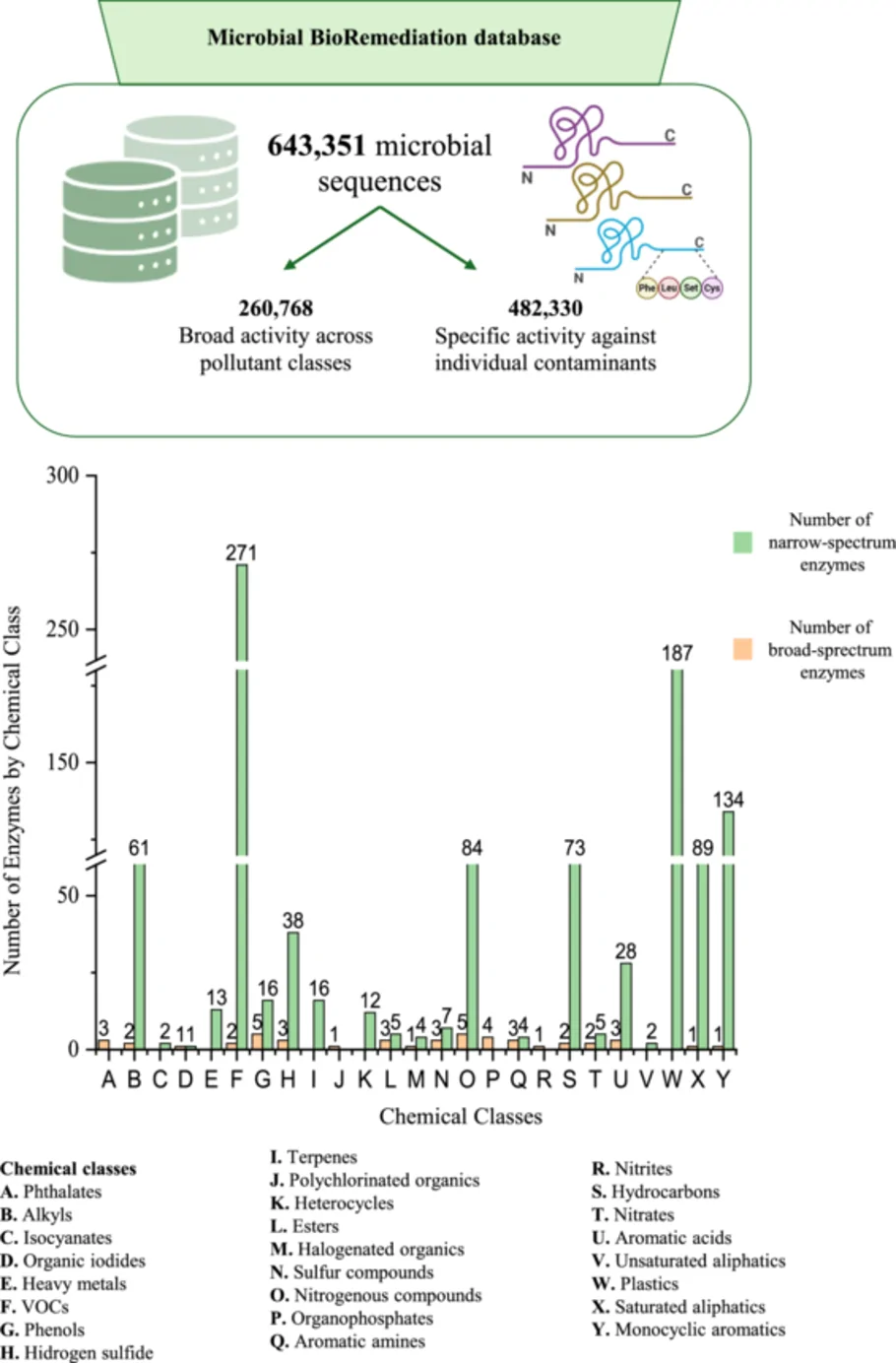

## 1 Introduction

Environmental pollution is a main global challenge with significant impacts on biodiversity, ecosystems and human health (Cheng et al. 2022; Singh et al. 2023; Bodor et al. 2024; Sanaullah et al. 2020). The primary sources of contamination are related to industrial, agricultural and urban activities (Gaur et al. 2020; Astuti 2015; Zalidis et al. 2002), which release into the environment organic compounds such as, polycyclic aromatic hydrocarbons, chlorinated solvents and pharmaceutical residues (Gomersall et al. 2025; Gao et al. 2025) and inorganic compounds including heavy metals (Alharbi et al. 2025). These pollutants, often persisting in the environment, impair soil fertility and water and air quality (Soleimani et al. 2023; Colborne et al. 2019), causing toxic effects, both acute and chronic, on living organisms (Cheng et al. 2022).

In this context, increasing anthropogenic pressure and climate change require developing effective and sustainable environmental remediation strategies. Over the past decade, microbiological studies have identified microorganisms accomplished of degrading or reclaiming the environment from pollutants such as plastics, heavy metals, and hydrocarbons (Yoshida et al. 2016; Hussein et al. 2024; Das and Chandran 2011). Thanks to the growing scientific literature on the subject, bioremediation, which exploits microorganisms, fungi, and plants to degrade pollutant compounds (Lokesh et al. 2023; Azubuike et al. 2016), has emerged as a promising biotechnological alternative with less impact on ecosystems than conventional solutions such as incineration and recycling, which tend to release additional pollutants into the environment (Idumah and Nwuzor 2019; Agnello et al. 2016). Despite advances in bioremediation, scientific research is still focused on a few bacterial species, limiting the understanding of metabolic diversity in pollutant degradation processes. Furthermore, although there are some specialized databases on individual categories of environmental pollutants (Gambarini et al. 2022; Buchholz et al. 2022; Gan and Zhang 2019), there is a lack of a comprehensive resource of all the genetic background involved in microbial bioremediation processes. This fragmentation underscores the need to comprehensive and curated genomic resources capable of representing the genetic potential of bacterial communities involved in the degradation of different pollutants.

To fill these gaps, this study presents the *Microbial BioRemediation* (*MBR*) database, which compiles genetic data on bacterial bioremediation. Particularly, it catalogues amino acid sequences involved in pollutant degradation, supporting both microbial strain characterization and the analysis of complex communities to identify bacteria with bioremediation potential. This tool aims to enable the design of large‐scale, next‐generation remediation strategies based on a broad understanding of the genetic basis of bioremediation and thus represents a crucial resource for future advances in this field.

## 2 Materials and Methods

### 2.1 Database Creation and Curation

The *MBR* database was constructed by collecting publicly available amino acid sequences of bacterial enzymes involved in the biodegradation of environmental pollutants. Relevant pollutant classes and compounds were identified through a literature review, and enzymatic reactions were retrieved from the Rhea database (Bansal et al. 2022), as well as from specialized resources focused on plastic degradation, including PlasticDB (Gambarini et al. 2022), PMBD (Gan and Zhang 2019), and PAZy (Buchholz et al. 2022).

For each function, EC numbers, reaction directionality, and cross‐references were extracted. Protein sequences were obtained from UniProt (Swiss‐Prot and TrEMBL) (Bateman et al. 2021) and RefSeq, using Rhea IDs or EC numbers as query terms. When necessary, additional sequences were retrieved from the same specialized databases and manually validated. All sequences were filtered to include only bacterial entries and were annotated with EC numbers, GO terms, and pathway metadata. The final database was formatted for compatibility with BLAST, DIAMOND, and METAnnotatorX2 (Milani et al. 2021).

### 2.2 Genomic Analyses

To genomically validate the *MBR* database, a comparative analysis was performed on publicly available genome sequences of bacterial genera known from scientific as encoding enzymes potentially involved in pollutant degradation. Specifically, for each genus, the genome sequence of reference bacterial strains was downloaded from the National Centre for Biotechnology Information (NCBI). A Basic Local Alignment Search Tool (BLASTp) protein analysis (e−value cutoff of 1e^‐100^) was then performed against the *MBR* database.

### 2.3 Metagenomic Analyses

Datasets corresponding to shotgun metagenomic samples of contaminated soil, river water, industrial wastewater and faeces were downloaded via the NCBI SRA repository. In detail, the obtained fastqs were filtered for quality using fastq‐mcf software (average quality score > 20 and read length > 100 bp (Viglioli et al. 2024). Subsequently, METAnnotatorX2 was used to obtain taxonomic and functional profiles from the metagenomic datasets (Milani et al. 2021). Specifically, for this study, the *MBR* database was integrated into METAnnotatorX2, allowing the precise identification of bacterial pollutant‐degrading functions. This analysis was applied to metagenomic datasets with the following parameters: 5,000,000 reads for metabolism assignment (‐‐query‐cover 80, ‐evalue 1×10^−8^ and ‐‐max‐target‐seqs. 1) (Milani et al. 2025). In addition, METAnnotatorX2 software (Milani et al. 2021) was used to obtain taxonomic profiles of the pool of reads associated with the bioremediation function identified in the datasets. All statistical analyses were performed using R software (version 4.4.3). Principal Coordinates Analysis (PCoA) based on Bray‐Curtis dissimilarities was applied to evaluate global differences in the predicted biodegradation functional profiles across samples.

## 3 Results and Discussion

### 3.1 Database Construction and Content

#### 3.1.1 Curation of Pollutant Classes and Relevant Enzymatic Pathways

The *MBR* database aims to represent a comprehensive and functionally informative resource regarding microbial bioremediation. The first step in the establishment of this database (Figure 1) involved the systematic identification and classification of chemical classes of environmental pollutants potentially subject to microbial bioremediation processes. To this end, we performed an extensive review of the scientific literature focused on the most recurrent contaminants in the environments and their documented biological degradability. Based on the information gathered, a system was developed based on two types of classification: a broader classification based on chemical classes and a narrow one based on individual pollutant compounds, to standardize the categorization of the chemical diversity of pollutants and to facilitate the association between functional groups and microbial enzymatic activities potentially involved in their degradation. The broader classification consists of 25 main chemical classes (Figure 2A) (Table S1), selected according to criteria of ecotoxicological impact and availability of microbiological data (Song et al. 2025a; Han et al. 2024; Kakimoto et al. 2024; Pakar et al. 2025; Zhang et al. 2025; Li et al. 2021; Yu et al. 2025; Liang et al. 2025a, 2025b; Jiang et al. 2025; Li et al. 2025a; Guerra 2001; Liao et al. 2025; Skogeng et al. 2025; KISHI et al. 2025; Rebelo et al. 2023; Othman et al. 2025; Grung et al. 2022; Samkampang et al. 2025; Mousavi et al. 2025; Li et al. 2025b; Cao et al. 2025; HÃ¤ggblom 1992). The narrow classification includes 564 specific compounds that can fall into one or more main chemical classes (Figure 2A) (Table S1), for which at least one or more enzymatic reactions potentially associated with their microbial degradation have been identified and verified through experimental sources or validated biochemical databases.

**Figure 1:**
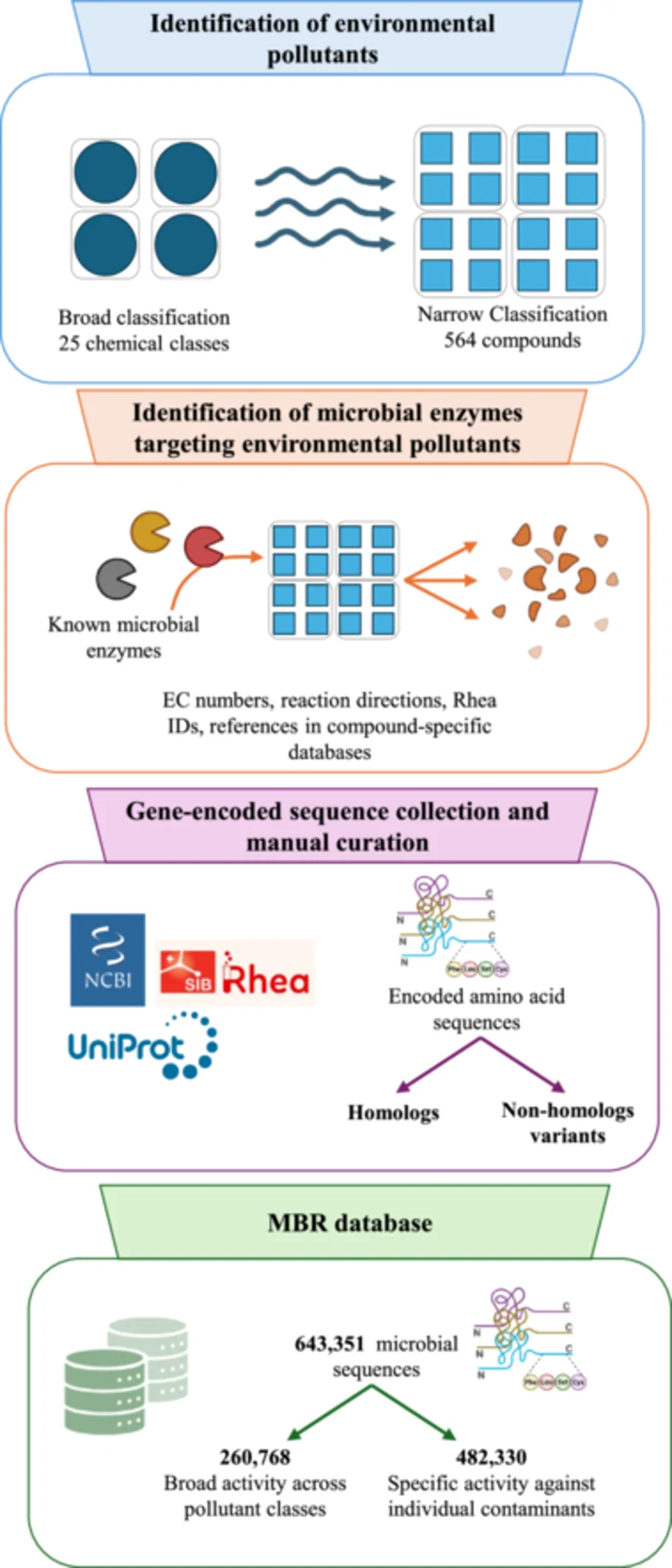
Workflow for the construction of the *MBR* database. The diagram illustrates the sequential steps followed to develop the *MBR* database. The figure was created using BioRender.com.

**Figure 2:**
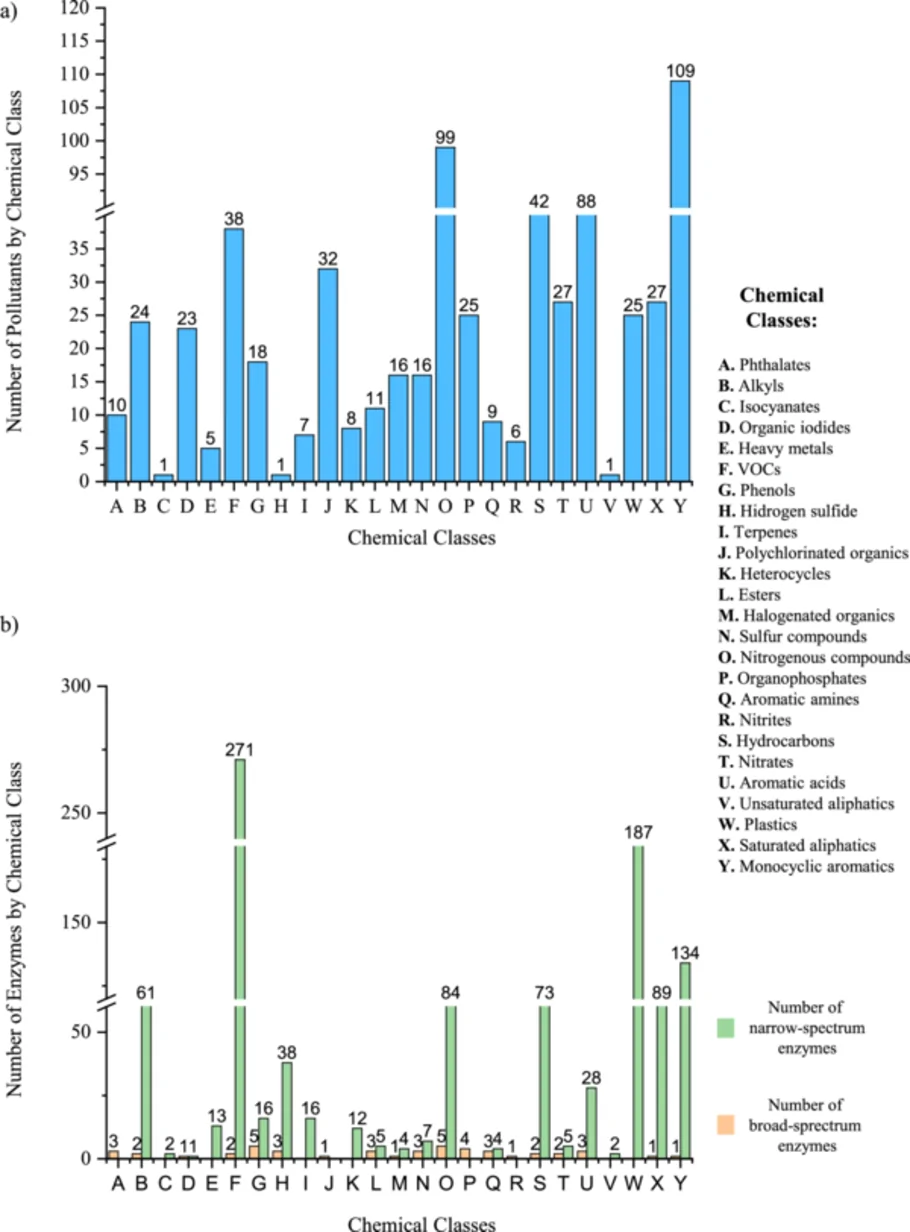
Structural Overview of the *MBR* database. Panel (a) displays the number of identified pollutant compounds grouped by chemical class. Note that some compounds are assigned to multiple chemical classes due to their structural or functional characteristics. Panel (b) shows the number of broad‐spectrum (pink) and narrow‐spectrum (green) enzymes identified for each chemical class. A single enzyme can act on more than one pollutant compound or chemical class, depending on its range of substrates.

Subsequently, for each compound, information was gathered on the bacterial enzymes involved in their degradation. This was done through a detailed analysis of curated biochemical databases such as the Rhea database (Bansal et al. 2022) along with other available public databases specializing in the microbial degradation or bioremediation of plastic pollutants (Gambarini et al. 2022; Buchholz et al. 2022; Gan and Zhang 2019), hydrocarbons (Wang et al. 2023) and metals (Pal et al. 2014). To ensure maximum coverage, publicly available databases specific to certain compound classes were also integrated. Specifically, curated resources for plastics (Gambarini et al. 2022; Buchholz et al. 2022; Gan and Zhang 2019), hydrocarbons (Wang et al. 2023), and metals (Pal et al. 2014) were screened to collect additional amino acid sequences not available in generalist biochemical repositories. In addition, the EC number, reaction direction, Rhea ID, and references in compound‐specific databases were extracted when available. This approach made it possible to generate a precise mapping of the relevant microbic enzymatic activities, which form the functional infrastructure of the *MBR* database, improving traceability between pollutant, chemical class, enzyme, and molecular function.

#### 3.1.2 Gene Collection and Sequence Curation

A thorough data collection and integration process was carried out to incorporate bacterial amino acid sequences related to microbial biodegradation into the *MBR* database (Figure 1). The sequences were categorized into enzymes that degrade entire classes of compounds (broad spectrum enzymes) (Figure 2B), and enzymes that target specific contaminants (narrow spectrum enzymes) (Figure 2B). Specifically, in the broad spectrum enzyme class, for 19 of the 25 main chemical classes, we identified amino acid sequences associated with enzymes acting non‐specifically on compounds falling into each of these classes (Table S2). In addition, Enzyme Commission (EC) numbers associated with broad enzymatic biodegradation reactions relevant to each class were identified (when available) to enhance accuracy in collecting the corresponding sequences. Subsequently, these EC numbers were used to query the NCBI RefSeq database, applying a filter for organisms belonging to the bacteria domain. For each amino acid sequence thus identified, its functional coherence concerning the degradation process of interest was verified based on the annotation and associated scientific literature. Remarkably, it was impossible to locate generic enzymes only for the following classes: Isocyanate Compounds, Heavy Metals, Terpenes, Heterocyclic Compounds, Plastics, and Unsaturated Aliphatics.

In the narrow spectrum enzyme class of the *MBR* database, amino acid sequences associated with the degradation of individual pollutant compounds previously identified (Table S3) were integrated by mapping each biochemical reaction catalogued in the Rhea database to the relevant UniProt identifiers (Bateman et al. 2021), including both Swiss‐Prot and TrEMBL entries, to ensure a broad and balanced coverage of the functional diversity of the microbial communities in the database.

In cases where microbial biodegradation functions were not linked to any Rhea entries or where Rhea reactions did not yet have associated protein sequences in UniProt (Bateman et al. 2021), the identification, selection, and integration of sequences was conducted using the same approach, based on publicly available databases specific to certain compound classes (Gambarini et al. 2022; Buchholz et al. 2022; Gan and Zhang 2019; Wang et al. 2023; Pal et al. 2014) or EC number, as described for the broader classification of the database.

The chemical classes and pollutant compounds for which no bacterial amino acid sequences potentially involved in the relevant biodegradation processes could be identified are listed in Supporting Table S4.

Overall, the presented multilevel approach made it possible to build a solid and functionally informative genetic basis for the *MBR* database, which is useful for analyses of microbial communities and single strains.

#### 3.1.3 Metadata validation and manual curation

For each *MBR* database entry corresponding to a microbial enzymatic reaction involved in biodegradation or bioremediation processes, a table was constructed containing key information, including *MBR* database identification code, EC number, molecular function description, reference metabolic pathway, and associated GO ontologies (Table S5). This approach ensures that information associated with an enzyme reaction is complete and easily accessible. In addition, a Supporting file was generated containing the complete details of the GO families represented in the database (Table S6), which is useful for wide‐ranging functional analyses. All files are available for download on the project website (https://probiogenomics.unipr.it/cmu), including preformatted database formats optimized for the BLAST and DIAMOND alignment tools. Additionally, a web‐based BLAST interface has been implemented, enabling users to align their query sequences against the MBR database directly online (https://probiogenomics.unipr.it/mbr/). A version compatible with the metagenomic analysis software METAnnotatorX2 (Milani et al. 2021), which is also used in the example test analyses reported in the following sections, is also included. Finally, the website provides a Bash script designed for the functional analysis of genes (e.g. a gene set of a microbial strain). This tool enables the generation of a comprehensive report of detected biodegradation functions, facilitating the integration of the *MBR* database into customized bioinformatics pipelines. These tools allow users to easily perform comparative genomics and metagenomic analysis on environmental samples, with the possibility of identifying biodegradation functions at the level of microbial communities. The open format and compatibility with different analysis tools make the *MBR* database highly interoperable, extending its usefulness in all research contexts.

#### 3.1.4 Comparison With Existing Resources

To date, no comprehensive and unified database systematically catalogues the genetic and enzymatic diversity underlying microbial biodegradation or bioremediation across all main classes of environmental pollutants. Existing resources are typically specialized, focusing on individual pollutant categories, such as plastics (Gambarini et al. 2022; Buchholz et al. 2022; Gan and Zhang 2019), hydrocarbons (Wang et al. 2023) or heavy metals (Pal et al. 2014) and often include a limited set of functionally annotated sequences. In contrast, the *MBR* database offers a broad, curated, and functionally structured representation of microbial biodegradation or bioremediation potential across diverse contaminant classes.

The *MBR* database integrates a total of 736 unique bacterial amino acid sequences directly linked to experimentally validated or biochemically supported biodegradation functions. To enhance sensitivity and functional breadth, all known homologous sequences associated with these enzymes were also included, bringing the total to 643,351 sequences (Figure 1). Of these, 260,768 are associated with broad‐spectrum enzymes (Figure 1), while 482,330 correspond to narrow‐spectrum enzymes (Figure 1). It is important to note that narrow‐spectrum enzymes may still act on chemically similar substrates, meaning some sequences may overlap between those considered broadly acting and those targeting specific contaminants. Additionally, for 69 compounds, up to 113 non‐homologous gene sequences have been integrated (Table S7), each presumed to encode enzymes acting on distinct chemical bonds within the same molecule. This inclusion ensures a more comprehensive representation of microbial metabolic versatility and redundancy in degradation mechanisms.

A comparative analysis was performed against multiple publicly available databases focused on microbial biodegradation or bioremediation. These included resources specific to plastic pollutants, PlasticDB, PMBD, PAZy (Gambarini et al. 2022; Buchholz et al. 2022; Gan and Zhang 2019), as well as repositories addressing other contaminant categories, such as the HMDB for aromatic hydrocarbons (Wang et al. 2023), and BacMet for metals (Pal et al. 2014). An additional resource is mibPOPdb, which is a manually curated database of microbial strains with documented potential for bioremediation in human‐associated environments (Ngara et al. 2022). Unlike the MBR database, mibPOPdb is restricted to strain‐level data, primarily targets persistent organic pollutants (POPs), and lacks support for large‐scale genomic or metagenomic analyses. As highlighted, no alternative comprehensive and unified database exists, thus preventing a direct comparison. As summarized in Table 1, the results demonstrate that while these currently available resources offer detailed insights into specific compound classes, the *MBR* database provides the most extensive and integrated collection of bacterial biodegradation genes currently available, encompassing a wider spectrum of enzyme families.

**Table 1:** Comparison of public and *MBR* databases in terms of annotated amino acid sequences.

Chemical classes	*MBR*	PlasticDB	PMBD	PAZy	HMDB	BacMet^a^
Plastics	96,340	228	8280	255	X	X
Alkyl compounds	17,528	X	X	X	X	X
Aromatic acid	17,784	X	X	X	X	X
Aromatic amines	6	X	X	X	X	X
Aromatics monocyclics	15,498	X	X	X	X	X
Esters	1683	X	X	X	X	X
Halogenated compounds	2	X	X	X	X	X
Heavy metals	5285	X	X	X	X	485
Heterocyclic compounds	1267	X	X	X	X	X
Hydrocarbons	26,308	X	X	X	1195	X
Hydrogen sulphide	113,037	X	X	X	X	X
Isocyanate compounds	4	X	X	X	X	X
Nitrates	5833	X	X	X	X	X
Nitrites	19011	X	X	X	X	X
Nitrogen compounds	52,184	X	X	X	X	X
Organic iodide compounds	14	X	X	X	X	X
Organophosphorus compounds	14,543	X	X	X	X	X
Phenolic Compounds	1667	X	X	X	X	X
Phthalatic compounds	19	X	X	X	X	X
Polychlorinated organic compounds	80	X	X	X	X	X
Saturated aliphatic	30,680	X	X	X	X	X
Sulphur compounds	12,080	X	X	X	X	X
Terpenes	40	X	X	X	X	X
Unsaturated aliphatics	864	X	X	X	X	X
Volatile organic compounds	194,832	X	X	X	X	X

*Note:* “X”: denotes data not available or not applicable for the pollutant class in the respective database.

^a^ BacMet database does not specifically focus on bacterial sequences involved in heavy metal bioremediation processes but rather includes a broad spectrum of bacterial sequences associated with metal resistance genes.

Notably, the *MBR* database includes a higher number of homologous sequences per enzymatic function than any of the compared databases (Table 1), enhancing the resolution and accuracy of functional annotation in metagenomic and genomic datasets. This level of enrichment facilitates the identification of biodegradation pathways in both individual strains and complex microbial communities. Overall, the *MBR* database represents a substantial advancement over existing resources, providing a unified, curated, and functionally structured database optimized for identifying and analysing microbial biodegradation pathways in isolated strains and complex environmental communities.

### 3.2 Applications of the *MBR* Database

#### 3.2.1 Validation of the *MBR* Database for Genomics Applications

To assess the utility of the *MBR* database for genome‐based screening of biodegradation potential, we performed a comprehensive functional analysis as a test case, using representative genomes corresponding to species of five bacterial genera widely recognized for their environmental relevance in pollutant degradation: *Alcanivorax* [number of genomes analyzed (*n*) = 9], *Pseudomonas* (*n* = 360), *Rhodococcus* (*n* = 43), *Sphingomonas* (*n* = 176), and *Cupriavidus* (*n* = 16) (Table S8). These taxa were selected based on their known ability to degrade or bioremediate the environment from a wide range of pollutants, including hydrocarbons, plastics, heavy metals, and aromatic compounds (Li et al. 2019; Kadri et al. 2022; Cao et al. 2022; Medić and Karadžić 2022; Song et al. 2025b; Wilkes and Aristilde 2017; Nazari et al. 2022; Chen et al. 2022; Yan et al. 2024; Zhao et al. 2017; Zhou et al. 2016; Panthi et al. 2024; Ridene et al. 2023; Kuppusamy et al. 2016). A set of five biodegradation functions was consistently detected across all 604 genomes analysed, regardless of genus (Table S9). These core functions, MBR_686, MBR_709, MBR_87, MBR_49, and MBR_228, are putatively involved in the degradation of catechol, Diisononylphthalate (DiBP), hydrogen sulphide, hydroquinone, and acetaldehyde, respectively (Figure 3). Their ubiquitous presence suggests the existence of a conserved biodegradative potential across phylogenetically distant taxa and underscores the sensitivity of the *MBR* database in detecting broadly distributed biodegradation capabilities. Beyond the core set, some genus exhibited distinctive profiles of presumably narrow‐spectrum functions, including several functions uniquely associated with individual genera (Figure 3). For example, only *Alcanivorax* genomes contained MBR_346, a function putatively involved in methanol degradation, aligning with the known specialization of this genus in marine hydrocarbon metabolism (Cao et al. 2022). Likewise, only *Rhodococcus* genomes harbored MBR_407, associated with isoprene degradation, and only *Cupriavidus* genomes included MBR_47, putatively involved in the degradation of polyhydroxybutyrate (PHB), a biodegradable plastic.

**Figure 3:**
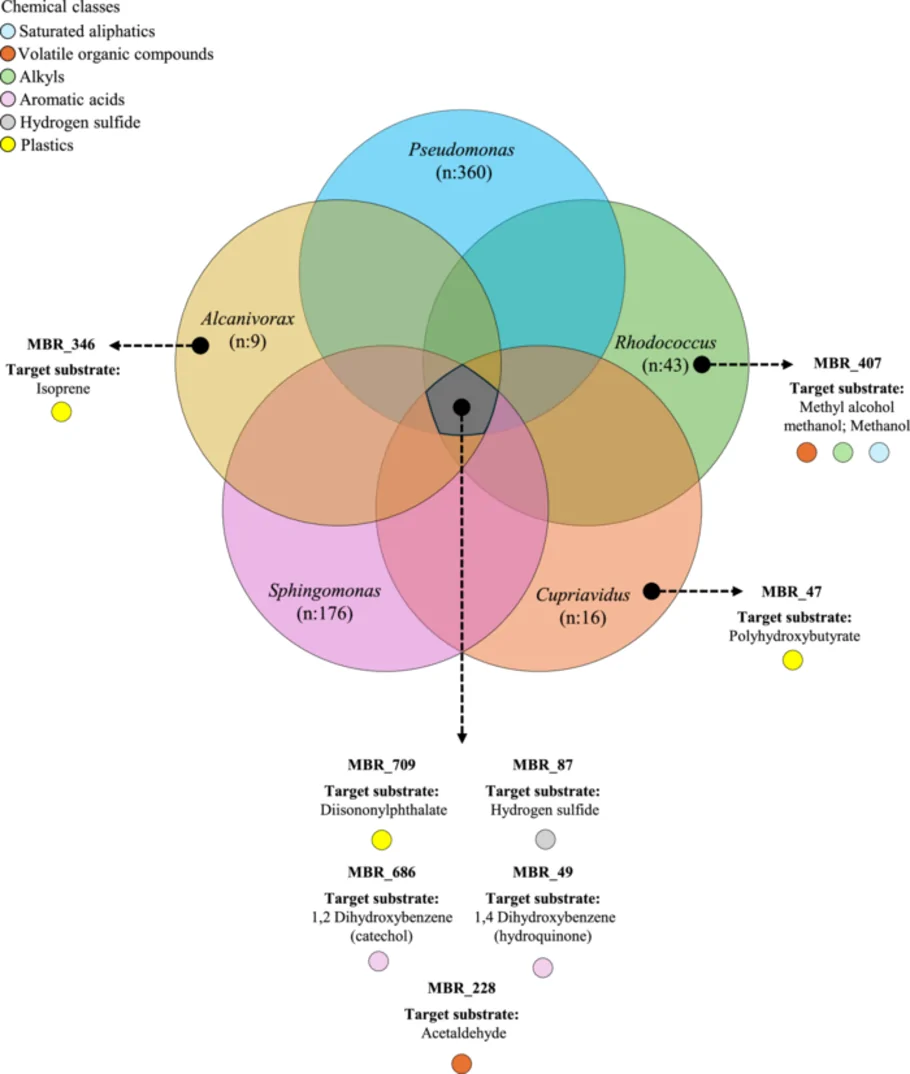
Biodegradation functions in bacterial genomes. The Venn diagram represents the distribution of enzyme functions integrated into the *MBR* database among five bacterial genera. Each circle corresponds to one genus and the number of analyzed genomes for each bacterial genus (n) is indicated next to the genus name. The diagram highlights both the core genome (functions shared across all five genera) and lineage‐specific functions (unique to individual genera). For each function, the chemical class to which the potential target of enzyme action belongs is color‐coded, and the target′s name is also reported.

This genome‐wide analysis confirms the capability of the *MBR* database to retrieve both conserved and lineage‐specific biodegradation functions, demonstrating its value for high‐resolution functional profiling of microbial genomes. By enabling the identification of pollutant‐specific pathways and highlighting microbial taxa potentially involved in key environmental degradation processes, the *MBR* resource offers a robust platform for microbial strain selection and the rational design of targeted bioremediation strategies. It is important to note, however, that this analysis represents a preliminary computational validation of the database; future steps should include detailed protein domain analysis to confirm functional annotations, as well as in vitro experiments to verify the actual biodegradation capacity of candidate strains.

To benchmark the performance of the *MBR* database against existing resources, we performed a comparative analysis using five representative genomes, each corresponding to one of the five bacterial genera previously analyzed (GCF_000009365.1, GCF_000219215.1, GCF_000412675.1, GCF_017068395.1, GCF_016027095.1). Each genome was screened using the *MBR*, PlasticDB, and HMDB databases (20, (Gambarini et al. 2022; Wang et al. 2023). PlasticDB and HMDB were selected as the only biodegradation databases among those considered that provided a downloadable multi‐FASTA file suitable for BLAST‐based analysis. The *MBR* database consistently identified a larger number of biodegradative functions than PlasticDB and HMDB databases (Table S10), highlighting its superior functional and chemical coverage and its suitability for high‐resolution genome mining and environmental metabolic profiling.

#### 3.2.2 Validation of the *MBR* Database for Metagenomics Applications

To validate the predictive power of the *MBR* database within complex microbial communities, we applied it to functionally publicly available metagenomic datasets from contaminated environments, based on the premise that bacteria naturally inhabiting these ecosystems may have evolved or horizontally acquired genes associated with pollutant degradation. A total of 20 public metagenomic datasets were selected and analyzed, including equal numbers from plastic‐contaminated soils (Liu et al. 2023), hydrocarbon‐contaminated soils (PRJNA1099207), plastic‐polluted river water samples (Zhou et al. 2022), and industrial wastewater (Lee et al. 2023) (Table S11).

Using METAnnotatorX2 software (Milani et al. 2021) with the MBR database as reference, microbial biodegradation functions were detected in all datasets (Figure 4) (Table S12). A total of 419 narrow‐spectrum enzymatic functions and 32 broad‐spectrum functions were identified across all datasets.

**Figure 4:**
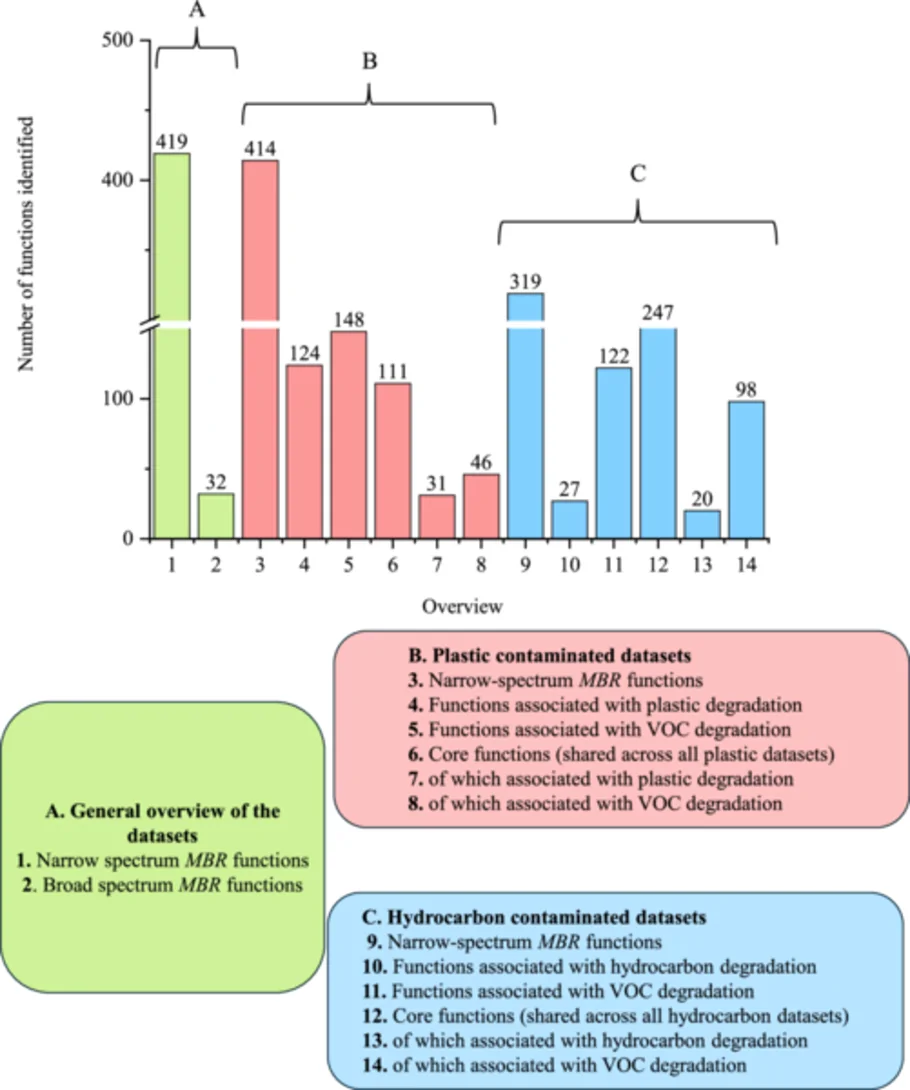
Functional profiling of metagenomic datasets from polluted environments using the *MBR* database. The bar plot illustrates the functional annotation results obtained from metagenomic datasets derived from four types of contaminated environments. Bars are color‐coded by pollutant class and grouped by dataset type, depicting the detection of biodegradation functions across different functional categories. The figure includes a general overview of narrow‐ and broad‐spectrum *MBR* functions identified across all datasets, followed by detailed profiling of plastic‐ and hydrocarbon‐contaminated datasets.

More detailed analyses were conducted on soil and river water samples contaminated by plastics (Table S13), as well as on hydrocarbon‐contaminated soils (Table S14), due to the greater certainty regarding the predominant pollutant classes in these environments. In contrast, due to the complex and variable nature of contaminants in the industrial wastewater samples, a targeted interpretation of specific biodegradation functions in these datasets was not conducted.

Specifically, in plastic‐contaminated soils and rivers (Figure 4) (Table S13), 124 enzymatic reactions specifically linked to plastic degradation pathways were identified. Additionally, we detected 148 functions associated with the degradation of volatile organic compounds (VOCs), common co‐contaminants in plastic‐polluted environments (Isaacson et al. 2025). A core set of 111 functions was consistently found across all plastic‐contaminated soil and river samples, including 31 plastic‐specific and 46 VOC‐related functions, indicating a conserved microbial potential for both primary and associated pollutant degradation (Table S13) (Figure S1).

To further investigate the functional differences between plastic‐contaminated soils and river water samples, we performed a Principal Coordinates Analysis (PCoA) based on Bray‐Curtis dissimilarity of the predicted enzymatic profiles (Table S13). The ordination revealed a partial separation between soil and river samples, which was statistically supported by a PERMANOVA test (*F* = 76.935, *R*² = 0.9058, *p* = 0.011), suggesting environment‐specific functional signatures, while also supporting the existence of a shared core of biodegradation functions across these environments. These results are provided as Supporting Information (Figure S2).

Similarly, functions profiled in hydrocarbon‐contaminated soils included 27 hydrocarbon‐specific functions (20 of which belonged to a shared core set), alongside 122 VOC‐degrading functions (98 in the core set) (Figure 4) (Table S14) (Figure S3). This highlights the metabolic versatility of microbial communities in these environments, reflecting the intertwined degradation pathways of hydrocarbons and VOCs.

Overall, these results obtained by the test case datasets confirm the utility of the *MBR* database as a tool for assessing the functional potential of microbial communities in polluted ecosystems. Nevertheless, as this analysis is based on predicted functional annotations, subsequent validation steps, including domain‐level protein analysis and experimental confirmation of biodegradative activity, are essential to strengthen the reliability of these inferences and fully translate metagenomic predictions into environmental applications.

#### 3.2.3 Application of the *MBR* Database to Host‐Associated Microbiomes: An Emerging Frontier

Applying the *MBR* database to host‐associated microbiomes opens new perspectives in understanding how environmental exposure to contaminants may influence microbial function in the gut. In this analysis, we used the *MBR* database to functionally profile 21 fecal samples, including 11 from beef cattle (Legrand et al. 2025), as well as 10 from adult humans of which five living in rural setting in a developing country (Obregon‐Tito et al. 2015), and five from adults living in urban industrialized settings (Obregon‐Tito et al. 2015) (Table S11). These samples represent ideal test cases as models for investigating the interaction between environment, diet, and microbiota under conditions of varying contaminant pressures.

In beef cattle, 304 distinct *MBR* biodegradation functions were identified across the 11 samples, with a core set of 121 functions consistently present in all (Table S15) (Figure 5A). The most represented chemical classes targeted by the core enzymatic set included volatile organic compounds (VOCs), plastics, hydrogen sulfide, nitrogen compounds, monocyclic aromatics, saturated aliphatics, and alkyl compounds. This diversity likely reflects the complex environmental and dietary exposures typical of agricultural settings, where cattle microbiomes encounter various pollutants related to farming practices and feed composition.

**Figure 5:**
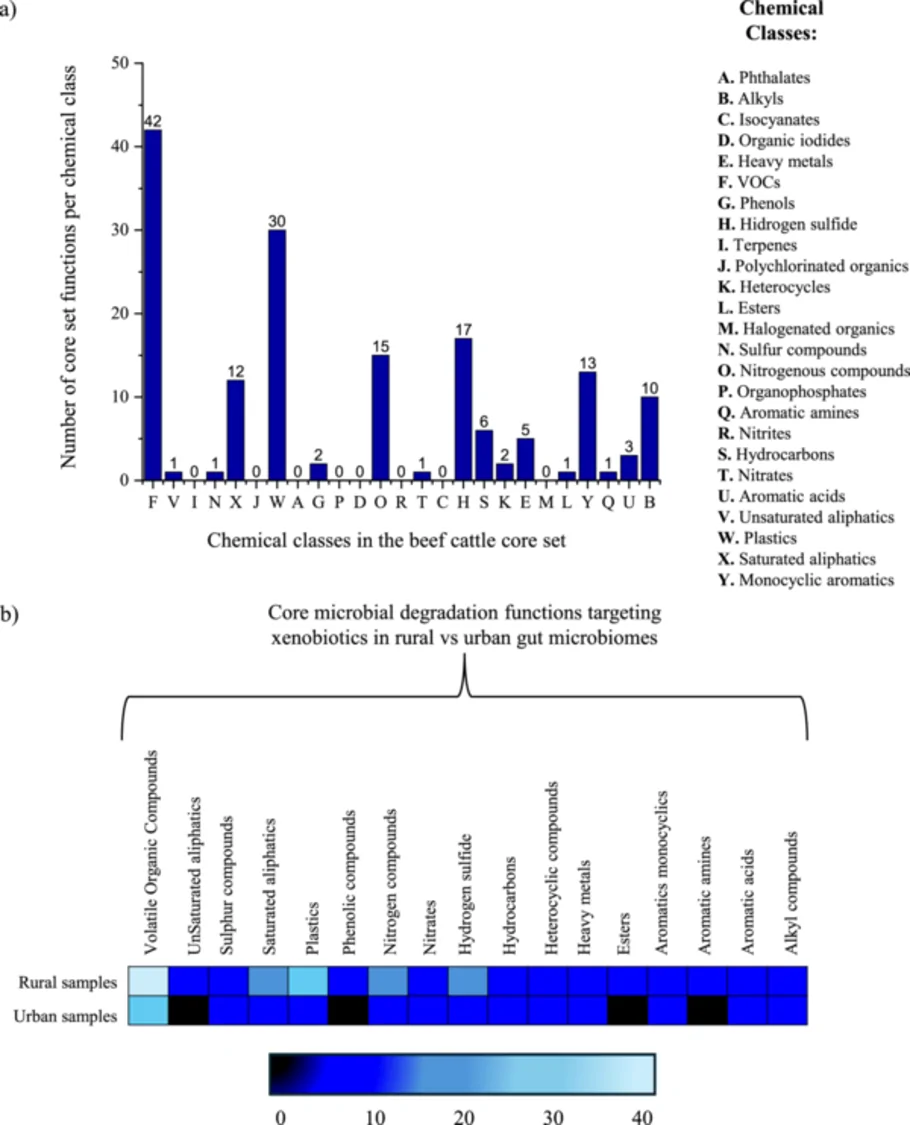
Distribution and comparison of enzyme functions by chemical class in the analysed datasets. Panel (a) shows the number of enzyme functions associated with each chemical class within the core set obtained from the analysis of the beef cattle datasets. Panel (b) dispays the heat map comparing the distribution of enzyme functions per chemical class between datasets from population living in rural setting in a developing country and urban populations. Each cell represents, with a colour code, the difference in the number of functions between the two groups for a specific chemical class.

To further explore potential applications for establishing associations between predicted bioremediation capacities and microbial taxa, metagenomic reads mapped to *MBR* functions in the animal datasets were taxonomically classified. Genera were then ranked based on their relative abundance within the mapped reads for each function, allowing us to identify those most likely contributing to the observed biodegradation capacities.

Among the core functions, the three most abundant narrow‐spectrum enzymes were potentially involved in the processing of Diisononylphthalate (DiBP), acetaldehyde and hydrogen sulfide. We evaluated which microbial genera were most associated with each function: *Alistipes*, *Flintibacter*, and *Pseudoflavonifractor* for DiBP biodegradation (Table S16); *Alistipes*, *Ruminococcus*, and *Clostridium* for hydrogen sulfide degradation (Table S16); and *Brachyspira*, *Alistipes*, and *Faecousia* for Acetaldehyde degradation (Table S16). While this analysis represents only an example of *MBR*'s potential applications, these findings support its use as a powerful tool to prioritize bacterial candidates for future experimental validation, strain isolation, and functional studies on the interaction between pollutants and animal farming.

Furthermore, comparative functional profiling of human host‐associated microbiomes from rural settings in developing countries and urban populations revealed notable differences in abundance and diversity of microbial biodegradation functions. It is important to clarify that the datasets from human living in rural setting in a developing country refer to the Tunapuco, which is a traditional agricultural community from the Andean highlands (Obregon‐Tito et al. 2015) whose diet is probably based on minimally processed foods and subject to less stringent control of environmental contaminants, whereas the “urban” datasets refer to urban‐industrialized communities in the United States (Obregon‐Tito et al. 2015), characterized by diets probably rich in highly processed foods and subject to strict contaminant regulations. Individuals from rural setting in a developing country exhibited a higher number of total *MBR* functions (207) and a more extensive core set (113) (Table S17) (Figure 5B) compared to urban counterparts (139 total and 60 core functions) (Table S17) (Figure 5B). This enhanced functional repertoire in indigenous populations microbiomes may be influenced by dietary and environmental factors typical of rural communities (Obregon‐Tito et al. 2015). Specifically, these populations living in developing countries often consume traditionally sourced, minimally processed foods that may not be subject to stringent contaminant controls, potentially leading to higher and more varied exposure to natural or environmental xenobiotics (Obregon‐Tito et al. 2015). These compounds often belong to chemical classes like VOCs, plastics, nitrogen compounds, and saturated aliphatics, all among the most represented classes in the indigenous core biodegradation functions.

While the presented results aim solely to demonstrate possible uses of the *MBR* database, the latter proves to be a valuable tool for elucidating how environmental exposures shape the biodegradation potential of host‐associated microbiomes. By enabling detailed functional and correlated taxonomic profiling, it supports future investigations into microbial adaptations to contaminants, with important implications for both host health and environmental bioremediation strategies.

Nonetheless, as with the previous applications of the *MBR* database, the predictions generated here are based on sequence‐level functional inferences, thus further validation steps, including protein domain analysis and in vitro experimentation, are necessary to confirm the functional activity of the implicated microbial taxa and enzymes.

## 4 Conclusions

The *Microbial BioRemediation* database represents a significant advancement in the field of environmental genomics and metagenomics, providing a comprehensive, curated and functionally structured resource for the study of microbial biodegradation of major environmental contaminants. To date, the *MBR* database integrates over 643,351 amino acid sequences associated with documented or predicted enzymatic functions in the degradation of 564 pollutant compounds belonging to 25 chemical classes. This infrastructure enables high‐resolution analysis of biodegradation potential in both isolated microbial genomes and complex communities through metagenomic approaches.

The database has proven effective in identifying core functions shared between phylogenetically distant microbial taxa, as well as specific functions associated with microbial genera or particular ecological niches. These capabilities make the *MBR* database a strategic tool for the initial selection and functional screening of candidate bacterial strains for bioremediation applications. However, it is important to emphasise that in vitro experimental confirmation of the predicted biodegradative capabilities remains essential to validate and translate this genomic and metagenomic evidence into effective biotechnological interventions. The use of the database in non‐environmental contexts, such as host‐associated microbiomes, has also revealed its potential in environmental medicine, showing how exposure to contaminants can shape the metabolic function of the microbiota. This approach opens new avenues for studies on the relationship between health, diet and environmental exposure, with implications also for personalised interventions based on functional modulation of the microbiome.

The database is freely accessible online (https://probiogenomics.unipr.it/cmu) and includes a user‐friendly interface with integrated BLAST functionality (https://probiogenomics.unipr.it/mbr/), enabling investigators to directly query their own sequences against the curated biodegradation dataset without requiring local installation. This feature broadens its applicability, facilitating rapid and accessible functional screening for a wide range of users.

Future developments of the *MBR* database aim to further expand its scope, with the integration of HMM models for protein domain prediction to improve the accuracy of functional assignments by better distinguishing homologous sequences with different substrate specificities. Moreover, efforts will be made to mitigate potential taxonomic bias arising from the inclusion of homologous sequences by implementing redundancy reduction strategies such as clustering and taxonomic normalization, aimed at improving genus‐level balance while maintaining comprehensive functional coverage. The use of artificial intelligence techniques will support the automatic prediction of new enzymatic functions and the annotation of uncharacterized sequences, potentially enabling the discovery of novel biodegradation pathways. In addition, the inclusion of data on non‐coding RNAs involved in biodegradation and the design of synthetic metabolic pathways will provide a more comprehensive systems‐level understanding of microbial degradation processes, facilitating the prioritization of targets for experimental validation and strain engineering. These developments will make *MBR* not only a database, but a dynamic platform supporting systemic research on a global scale, which is essential to address future environmental challenges.

## Author Contributions

Data acquisition, Formal analysis, and Writing – original draft: S.P. Data curation: C.T., L.M., and G.A.L. Supervision: F.T., C.M., and M.V. Conceptualization: C.M. and M.V. Writing – review and editing: F.T., C.M., and M.V.

## Ethics Statement

The authors have nothing to report.

## Conflicts of Interest

The authors declare no conflicts of interest.

## Supporting information


**Supporting Figure 1:** Relative abundance heatmap of MBR core biodegradation functions across plastic‐contaminated river (blue triangles) and soil (brown triangles) samples.

Supporting Information

**Supporting Figure 2:** Principal Coordinates Analysis (PCoA) of predicted biodegradation functions in plastic‐contaminated soils and river water samples.

**Supporting Figure 3:** Heatmaps showing the relative abundance of MBR core biodegradation functions in hydrocarbon‐contaminated soil samples.

**Supporting Table 1:** Main chemical classes and pollutant compounds selected for the construction of the *MBR* database. **Supporting Table 2:** Bacterial amino acid sequences, integrated in the MBR database, associated with enzymes capable of acting non‐specifically on several compounds of the same chemical class. **Supporting Table 3:** Bacterial amino acid sequences, integrated in the MBR database, associated with enzymes capable of acting specifically on several pollutant or toxic compounds. **Supporting Table 4:** Chemical classes and pollutant compounds for which no putative bacterial amino acid sequences involved in biodegradation could be identified. **Supporting Table 5:** Detailed information for each MBR database entry corresponding to microbial enzymatic reactions involved in biodegradation of pollutant compounds. **Supporting Table 6:** GO families represented in the database. **Supporting Table 7:** Non‐homologous amino acid sequences integrated into the *MBR* database by contaminant. **Supporting Table 8:** Reference genomes of the genera Alcanivorax, Pseudomonas, Rhodococcus,Sphingomonas and Cupriavidus on which genomic analyses were performed. **Supporting Table S9:** Blastp analysis on the reference genomes of the genera *Alcanivorax, Pseudomonas, Rhodococcus,Sphingomonas and Cupriavidus*. **Supporting Table S10:** Comparative analysis of five representative bacterial genomes screened using the *MBR*, PlasticDB and HMDB databases. **Supporting Table 11:** Metagenomic datasets analysed in the study. **Supporting Table 12:** Detection of microbial biodegradation functions in environmental datasets using METAnnotatorX2 with the *MBR* database. **Supporting Table 13:** Detailed analysis of microbial biodegradation functions in plastic‐contaminated soil and river water samples using METAnnotatorX2 with the *MBR* database. **Supporting Table 14:** Detailed analysis of microbial biodegradation functions in hydrocarbon‐contaminated soil samples using METAnnotatorX2 with the *MBR* database. **Supporting Table 15:** Detailed analysis of microbial biodegradation functions in fecal samples from beef cattle using METAnnotatorX2 with the *MBR* database. **Supporting Table S16:** Taxonomic profiling of microbial genera associated with key biodegradation functions in animal‐associated metagenomes. **Supporting Table S17:** Comparative analysis of microbial biodegradation functions in human gut microbiomes from rural and urban populations using METAnnotatorX2 and the *MBR* database.

## Data Availability

Data available in article Supporting information.
