# Supplementary material for: Microbial BioRemediation Database: A Comprehensive Database of Genes Involved in Microbial Bioremediation Processes
Source: Microbiologyopen. 2026 Jan 27;15(1):e70215. doi: 10.1002/mbo3.70215 (PMC13750487; doi:10.1002/mbo3.70215)
Supplement: Supplementary file 1 [file MBO3-15-e70215-s001.pdf]

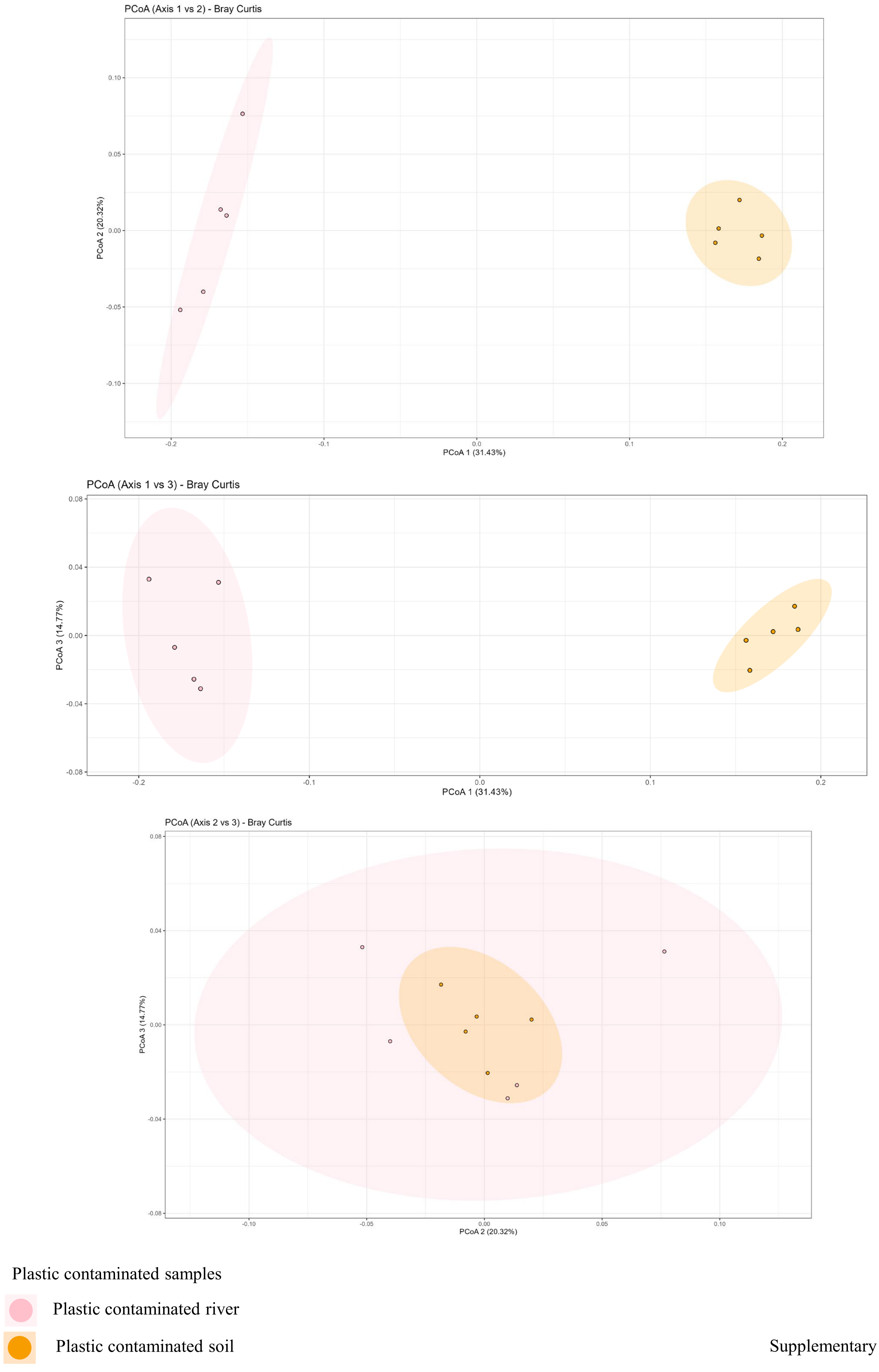

Supplementary figure 2
