# Supplementary material for: Microbial BioRemediation Database: A Comprehensive Database of Genes Involved in Microbial Bioremediation Processes
Source: Microbiologyopen. 2026 Jan 27;15(1):e70215. doi: 10.1002/mbo3.70215 (PMC13750487; doi:10.1002/mbo3.70215)
Supplement: Supplementary file 4 [file MBO3-15-e70215-s005.docx]

**Supplementary figure 1.** Relative abundance heatmap of MBR core biodegradation functions across plastic-contaminated river (blue triangles) and soil (brown triangles) samples. Each row represents a reaction, with color intensity indicating its abundance. Circles denote enzyme activity on volatile organic (dark green) or hydrocarbon (light green) pollutants.

**Supplementary figure 2.** Principal Coordinates Analysis (PCoA) of predicted biodegradation functions in plastic-contaminated soils and river water samples. Ordination based on Bray-Curtis dissimilarity of MBR functional profiles. The plot shows a partial separation between soil (orange) and river water (pink) samples, indicating distinct functional patterns, alongside a shared core of functions present in both environments.

**Supplementary figure 3.** Heatmaps showing the relative abundance of MBR core biodegradation functions in hydrocarbon-contaminated soil samples. Each column represents a specific enzymatic reaction or functional pathway, with color intensity indicating its relative abundance across samples. The first heat map shows enzymatic functions potentially involved in the degradation of hydrocarbon compounds; the second displays functions related to the degradation of volatile organic compounds (VOCs); the third illustrates enzymatic functions potentially involved in the biodegradation of both hydrocarbon pollutants and VOCs.
